# Dysmenorrhea and depressive symptoms among female university students: a descriptive study from Saudi Arabia

**DOI:** 10.1186/s41983-022-00542-1

**Published:** 2022-09-06

**Authors:** Deemah Alateeq, Lolwah Binsuwaidan, Leenah Alazwari, Maram Algarni, Maryam Al Hussain, Raghad Alzahrani, Reema Aljohani

**Affiliations:** Clinical Sciences Department, College of Medicine, Princess Nourah Bint Abdulrahman University, P.O. Box 84428, Riyadh, 11671 Saudi Arabia

**Keywords:** Dysmenorrhea, Depression, Female students, Menstrual cycle, Pain

## Abstract

**Background:**

Dysmenorrhea has significantly increased in prevalence. There is also evidence of the coexistence of dysmenorrhea and psychological disorders. This study aims to explore the prevalence of dysmenorrhea and investigate its correlation with depressive symptoms among Princess Nourah bint Abdulrahman University (PNU) students. All participants (*N* = 487) in this cross-sectional study provided sociodemographic data, menstrual and medical history, and completed the Patient Health Questionnaire (PHQ-9) scale and (working ability, location, intensity, days of pain, dysmenorrhea [WaLIDD]) scale on a self-administered online questionnaire.

**Results:**

The mean age of the females was 20.64 ± 2.38 years, and 40.7% were from health colleges. Severe dysmenorrhea requiring medical attention and pain killers or herbs was reported by 30.8% of the students. Significant predictors of severe dysmenorrhea were: younger age, earlier menarche, pain killers and herbs used for menstrual pain, a doctor visit for menstrual pain, and depression. In addition, significant protective factors of depression were: later menarche age, having a regular menstrual cycle, and longer duration.

**Conclusions:**

Students with severe dysmenorrhea have a higher risk of depression than other students. The findings stress the importance of awareness, education, a multidisciplinary approach to women's health, and early detection to prevent future complications.

## Background

Dysmenorrhea is common among adolescent women and involves colicky pain or cramping related to menstruation, usually localized in the lower abdomen but may extend to the back and thighs [1, 2]. There are two types of dysmenorrhea: primary dysmenorrhea, which is associated with a normal menstrual cycle; and secondary dysmenorrhea, which is related to an underlying pathological condition [3]. Very little was found in the literature on the question of the pathophysiology of primary dysmenorrhea. However, the most relevant explanation is due to the hyperstimulation of the uterine inner lining producing high amounts of prostaglandins. It is noticed that the Prostaglandins effects start once there is a drop in the Progesterone levels. Prostaglandins stimulate uterine contraction by increasing uterine tone. Another interesting finding is how vasopressin leads to ischemic pain by increasing the uterine contractility [4]. To date, several studies have investigated the link between psychological disorders and dysmenorrhea. Surprisingly, one study on rats showed that Prostaglandin levels was found to be high in plasma after 30 min of stress exposure [5]. This finding raise intriguing questions regarding the nature and extent of psychological disorders’ effect on women’s well-being.

Recent studies have shown that dysmenorrhea is widespread globally and locally. International studies show a prevalence estimate ranging from 56 to 80% [6, 7], whereas national studies have reported an incidence of 60.9–89.7% among university students in Saudi Arabia [2, 3, 8, 9]. Prior studies have reported that dysmenorrhea can affect women’s quality of life and mental status [10]. For example, a study conducted at King Saud University reported a dysmenorrhea prevalence of 80.1% among their students and further described the negative impact it had on their school performance, including decreased attendance and participation [2]. Moreover, 89% of the students in that study confirmed the negative influence dysmenorrhea had on their concentration [2]. A study in India also found a high prevalence of dysmenorrhea (71.96%) and showed that psychological symptoms, including depression, are significantly associated with dysmenorrhea [11]. Only a few studies regarding the prevalence of dysmenorrhea and its association with depression are available in the literature [6, 11, 12]. However, most of these were either international or did not utilize a validated dysmenorrhea tool [3].

Here we aim to confirm the association between psychological and gynecological health. This study explores the prevalence of dysmenorrhea and investigates its correlation with depression among Saudi university students.

## Methods

The sampling frame included female Princess Nourah bint Abdulrahman University (PNU) students from multiple colleges, which have 38,986 students. The sample was calculated using Raosoft software, and the required sample size was estimated at the 95-confidence level with an estimated 50% response distribution and a margin of error of ± 5%. The recommended sample size was 381. We had obtained 488 participants.

A pre-designed, anonymous, self-administered online questionnaire was utilized in this study. The questionnaire was handed to the head students at each college and distributed during February and March 2021. The study Institutional Review Board approval number (IRB-PNU: 20-0545). It was performed in accordance with the ethical principles stated in the Declaration of Helsinki. Written informed consent was acquired from all respondents.

The study followed a descriptive cross-sectional design based on a convenient nonprobability sampling technique. Inclusion criteria were: Female, Arabic-speaking PNU students above 17 years. Graduate and non-PNU students were excluded.

The questionnaire covered four domains: demographics, menstrual and medical history, depression, and dysmenorrhea. (1) Demographic characteristics included age, marital status, number of children, family’s monthly income, and college. The colleges included in the questionnaire are under four main faculties which are Health Sciences, Humanities, Sciences, and Social work colleges. Health Sciences collages years range from 5 to 6 years. Whereas the rest has an average of 4 years. (2) Menstrual and medical history included age of first menarche, menstrual cycle regularity, the use of painkillers and herbs for the menstrual pain, doctor visits for menstrual pain, and a history of gynecological disease and psychiatric disorder. (3) Depression was assessed by The Patient Health Questionnaire (PHQ-9), a well-known depression screening scale. It scores each of the nine DSM-5 criteria from “0” (not at all) to “3” (nearly every day) and has been validated for use in Arabic. The total score ranges from 0 to 27, categorized as mild, moderate, moderately severe, and severe depression [13, 14]. (4) Dysmenorrhea was assessed by working ability, location, intensity, days of pain, and dysmenorrhea (WaLIDD) score, which integrates features of dysmenorrhea, such as: number of anatomical pain locations; Wong–Baker pain range; the number of days of pain during menstruation; and frequency of pain too disabling to perform daily activities. Each of the tool’s items is scored between 0 and 3, and the final score ranges from 0 to 12 points, which is categorized as mild, moderate, and severe dysmenorrhea [15].

The data were described as means and standard deviations for the continuous variables and frequencies and percentages for the categorical variables. The histogram and the Kolmogorov–Smirnov statistical test of normality were used to assess the distribution of metric variables. Cronbach’s alpha test of internal consistency was used to assess the internal consistency of the questionnaire data. The bivariate Pearson’s product moments correlations test (*r*) was used to assess the correlation between continuously measured scores. The chi-squared (χ^2^) test was used to assess correlations between categorically measured variables. The WaLIDD and PHQ-9 scores were categorized into levels based on their author’s scoring methods, but the WaLIDD score was also dichotomized to characterize severe dysmenorrhea requiring medical attention and taking pain killers or herbs based on a cutoff of > 9 points [15]. Multivariate logistic binary regression analysis was used to assess the combined and individual associations between participants’ odds of having severe dysmenorrhea with their sociodemographic and menstrual characteristics. The multivariate generalized linear model with gamma regression was applied to regress the participants’ mean depression score against their sociodemographic and menstrual characteristics and dysmenorrhea risk scores. The associations between the predictor independent variables and the analyzed outcomes were expressed as odds ratios (ORs) and the associated 95% confidence intervals (CIs). The SPSS IBM statistical data analysis program version 20 was used for all statistical analyses and the alpha significance level was set at 0.050.

## Results

The sociodemographic characteristics of the study sample (487 participants) are shown in Table 1. The average age was 20.64 ± 2.38 years. Most of the participants were not married (95.9%) and did not have children (96.9%). More than one-third of the participants were from health colleges (40.7%), while the community college accounted for at least 10.9%. Regarding family monthly income, 46.2% reported having enough income, while 4.7% reported not having enough income and did not have savings.

**Table 1:** Descriptive analysis of the sociodemographic characteristics and menstrual and medical history (*n* = 487)

Item	Frequency (%)/mean ± SD
1. Age	20.64 ± 2.38
< 20 years	157 (32.2%)
20–24 years	307 (63%)
> 25 years	23 (4.7%)
2. College	
Health colleges	198 (40.7%)
Science colleges	148 (30.4%)
Humanities colleges	91 (18.7%)
Community college	50 (10.3%)
3. Marital status	
Married	20 (4.1%)
Not married	467 (95.9%)
4. Do you have children	
Yes	15 (3.1%)
No	472 (96.9%)
5. Family’s monthly income	
Enough with savings	194 (39.8%)
Enough	225 (46.2%)
Not enough	45 (9.2%)
Not enough and without savings	23 (4.7%)
6. Age of menarche	12.83 ± 1.64
< 11 years	88 (18.1%)
12–14 years	323 (66.3%)
> 15 years	76 (15.6%)
7. Is your menstrual cycle regular?	
Yes	326 (66.9%)
No	161 (33.1%)
8. Duration of menstrual cycle in days	6.53 ± 1.60
9. Do you use any painkillers for menstrual pain?	
Yes	233 (47.8%)
No	254 (52.2%)
10. Do you use any herbs for menstrual pain?	
Yes	237 (48.7%)
No	250 (51.3%)
11. Have you ever been to a doctor because of menstrual pain?	
Yes	93 (19.1%)
No	394 (80.9%)
12. Have you ever been diagnosed with a gynecological disease?	
Yes	42 (8.6%)
No	445 (91.4%)
13. Have you ever been diagnosed with a psychiatric disorder?	
Yes	54 (11.1%)
No	433 (88.9%)

The students’ menstrual and medical histories are also presented in Table 1. The average age of menarche was 12.83 ± 1.64 years. However, the categorized age of menarche onset showed that most had their first menses between 12 and 14 years of age (66.3%). Two-thirds of the participants reported having a regular menstrual cycle (66.9%) with a mean duration of 6.53 ± 1.60 days. Almost half of the participants used painkillers and herbs for menstrual pain (47.8% and 48.7%); however, only 19.1% reported that they had visited a doctor for menstrual pain. Regarding their medical history, only 8.6% of participants were diagnosed with a gynecological disease, and 11.1% were diagnosed with a psychiatric disorder.

The WaLIDD score was a reliable measure according to Cronbach’s alpha test (Cronbach’s alpha = 0.722). The mean total score was 7.13 out of 12 points with a standard deviation of 2.32 points, suggesting a moderate level of dysmenorrhea. Furthermore, dysmenorrhea levels ranged from mild (14.6%) to moderate (39.8%) and severe (45.6%). However, 30.8% of participants had severe dysmenorrhea requiring medical attention and pain killers or herbs.

As reported in Table 2, working ability was always or almost always affected by menstrual pain in 65.7% of the students. Most of the students had menstrual pain in their inguinal region (lower abdomen) (45.1%), and 35.6% had it in the lumbar region (lower back). In addition, 41.1% of students reported a pain severity of 4–6/10 on the Wong–Baker pain scale and 36.6% reported a pain severity of 8–10/10. Most of the students reported that the duration of their menstrual pain was 1–2 days (75.6%).

**Table 2:** Descriptive analysis of the dysmenorrhea WaLIDD score (*n* = 487)

Item	Frequency (%)
1) How much does menstrual pain affect your working ability?	
Does not affect	44 (9%)
Almost never	123 (25.3%)
Almost always	196 (40.2%)
Always	124 (25.5%)
2) Menstrual pain location (s)	
It is not painful	26 (5.3%)
Lumbar region (lower back)	335 (35.6%)
Thigh region	181 (19.3%)
Inguinal region (lower abdomen)	424 (45.1%)
3) Menstrual pain severity	
Does not hurt	17 (3.5%)
Hurts a little bit	92 (18.9%)
Hurts a little more—hurts even more	200 (41.1%)
Hurts a whole lot—hurts worst	178 (36.6%)
4) Days of menstrual pain	
0 days	15 (3.1%)
1–2 days	368 (75.6%)
3–4 days	88 (18.1%)
5 or more days	16 (3.3%)

Cronbach’s alpha test of internal consistency showed that the PHQ-9 was read and understood by the participants equally reliably (Cronbach’s alpha = 0.89). The scale total score mean was 12.05 out of 27 points with a standard deviation of 6.85 points. In general, the PHQ-9 scale categorizes depression into mild (28.3%), moderate (23.2%), moderately severe (19.7%), and severe (14.6%). The depressive symptoms noticed by the participants are presented in Table 3.

**Table 3:** Descriptive analysis of the PHQ-9 scale (*n* = 487)

Depressive symptoms	Mean ± SD
1. Little interest or pleasure in doing things	1.34 ± 0.95
2. Feeling down, depressed, or hopeless	1.56 ± 1.00
3. Trouble falling or staying asleep, or sleeping too much	1.69 ± 1.1
4. Feeling tired or having little energy	1.79 ± 0.95
5. Poor appetite or overeating	1.54 ± 1.06
6. Feeling bad about yourself or that you are a failure or have let yourself or your family down	1.29 ± 1.16
7. Trouble concentrating on things, such as reading the newspaper or watching television	1.15 ± 1.11
8. Moving or speaking so slowly that other people could have noticed. Or the opposite being so fidgety or restless that you have been moving around a lot more than usual	0.98 ± 1.08
9. Thoughts that you would be better off dead, or of hurting yourself	0.73 ± 1.07

The Pearson’s correlations test in Table 4 showed that the students’ mean WaLIDD score correlated significantly and positively with their PHQ-9 mean score (*r* = 0.401, *p* < 0.010). Moreover, the menstrual pain’s effect on working ability, the number of painful body locations, menstrual pain severity, and the number of menstrual pain days all correlated significantly and positively with the mean depression score (*p* < 0.010). In addition, the female students' age of first menstrual cycle correlated significantly and negatively with the mean depression score (*r* = − 0.105, *p* < 0.050).

**Table 4:** Bivariate correlations between dysmenorrhea, depression, and other variables

	PHQ-9 score	WaLIDD score	Working ability	Pain locations	Pain severity	Days of pain	Age	Length of cycle
WaLIDD score	0.401**	1						
Menstrual pain affects working ability score	0.371**	0.844**	1					
Menstrual pain locations score	0.302**	0.667**	0.347**	1				
Menstrual pain severity score	0.286**	0.865**	0.713**	0.410**	1			
Days of menstrual pain score	0.205**	0.541**	0.316**	0.153**	0.360**	1		
Age (years)	− 0.065	− 0.119**	− 0.081	− 0.051	− 0.111*	− 0.128**	1	
Length of menstrual cycle in days	− 0.070	0.098*	0.068	0.064	0.065	0.111*	− 0.083	1
Age of menarche	− 0.105*	− 0.022	0.005	− 0.093*	− 0.036	0.094*	− 0.010	− 0.029

**Correlation is significant at the 0.01 level (2-tailed). *Correlation is significant at the 0.05 level (2-tailed)

The WaLIDD subscale scores correlated significantly and positively with each other. The mean WaLIDD total score correlated significantly and negatively with their current age in years (*r* = − 0.119, *p* < 0.010), indicating that older females experienced significantly less severe dysmenorrhea. In addition, age correlated significantly and negatively with menstrual pain severity and duration, with older females experiencing slightly less intense menstrual pain and shorter menstrual pain duration (*p* < 0.050 and *p* < 0.010, respectively). Moreover, the age of menarche correlated negatively and significantly with the number of painful locations and days (*p* < 0.050). In addition, menstrual cycle length in days correlated positively and significantly with the WaLIDD score and the number of painful days (*p* < 0.050).

The multivariate logistic binary regression analysis of the female students’ odds of having severe dysmenorrhea (> 9 points on the WaLIDD scale) requiring medical attention and pain killers or herbs is displayed in Table 5. These data indicate that the age of menarche correlated significantly and negatively with the odds of experiencing severe dysmenorrhea, indicating that for each 1-year delay in the onset of puberty, the odds of having severe dysmenorrhea declined by 17.4% on average (*p* value = 0.016). Moreover, the analysis model showed that four variables were significant predictors of severe dysmenorrhea: pain killer use for menstrual pain, herb use for menstrual pain, need a doctor for was found to be significantly more inclined to have severe dysmenorrhea (4.95 and 1.82 times more) compared with those who do not use pain killers (*p* value < 0.001 and *p* value = 0.012, respectively). Females who needed to consult a doctor for their menstrual pain were significantly more likely to have severe dysmenorrhea (2.41 times more) compared with those who had not (*p* value = 0.002). In addition, females’ mean depression scores converged significantly and positively on their odds of severe dysmenorrhea (*p* value < 0.001). For each one-point rise in the PHQ-9 score, their odds of having severe dysmenorrhea rose by 12.1%.

**Table 5:** Multivariate binary logistic regression analysis of the students’ odds of severe dysmenorrhea

Items	Multivariate adjusted odds ratio (OR)	95% CI for OR		*p* value
		Lower	Upper	
(Intercept)	0.816	0.023	28.520	0.911
Age (years)	0.909	0.809	1.021	0.108
Has children	3.700	0.674	20.320	0.132
Sufficiency of family monthly income	1.221	0.927	1.608	0.156
Age of menarche (years)	0.830	0.712	0.966	0.016
Has a regular menstrual cycle	0.653	0.398	1.072	0.092
Duration of menstrual cycle (days)	1.081	0.938	1.246	0.285
Pain killer use for menstrual pain	4.948	3.012	8.128	< 0.001
Herb use for menstrual pain	1.818	1.143	2.891	0.012
Needs a doctor for menstrual pain	2.407	1.365	4.246	0.002
PHQ-9 mean score	1.121	1.079	1.166	< 0.001
Positive history of gynecological disease	0.493	0.198	1.229	0.129
Positive history of psychiatric disorder	0.808	0.373	1.746	0.587

Dependent variable: Severe dysmenorrhea (WALIDD score > cutoff of 9)

Generalized multivariate linear regression analysis with gamma regression was used to regress the female students’ PHQ-9 score against the other variables, which is displayed in Table 6. Females’ age of menarche correlated significantly but negatively with depression mean score (*p* value = 0.031). For every 1-year delay in puberty onset, their odds of having depression declined by 3.31% on average. In addition, female students with regular menstrual cycles measured significantly lower depression mean scores (15.8% less) compared with those with irregular menstrual cycles (*p* = 0.001). Menstrual cycle duration correlated significantly but negatively with depression mean score (*p* value = 0.028). For each 1-day increase in the duration of the menstrual cycle, their mean predicted depression score declined by 2.9%. In addition, having a positive history of a psychiatric disorder was found to be a significant predictor of depression (*p* < 0.001). Interestingly, the female students’ mean WaLIDD score correlated significantly and positively with their mean PHQ-9 score (*p* value < 0.001). For each one-point rise in the WaLIDD score, the depression score tended to rise by 9.4%.

**Table 6:** Generalized multivariate linear analysis of the depression mean score

Items	Multivariate adjusted risk rate (RR)	95% CI for RR		*p* value
		Lower	Upper	
Age (years)	0.999	0.975	1.024	0.947
Age of menarche (years)	0.967	0.938	0.997	0.031
Married	1.325	0.977	1.795	0.070
Regular menstrual cycle	0.842	0.758	0.936	0.001
Duration of menstrual cycle (days)	0.970	0.944	0.997	0.028
Pain killers used for menstrual pain	0.924	0.823	1.038	0.185
Needs a doctor for menstrual pain	1.117	0.977	1.276	0.105
Positive history of psychiatric disorder	1.443	1.232	1.690	< 0.001
WaLLID mean score	1.094	1.066	1.122	< 0.001

Dependent variable: PHQ-9 mean score. Estimating method: Maximum likelihood with gamma link

RR = Risk rate = nearly the same interpretation of odds ratio, if not the same

## Discussion

Dysmenorrhea is one of the most prevalent disorders affecting 90% of reproductive-age women [16]. In the current study, the WaLIDD scale was used to measure dysmenorrhea, which was a reliable tool with an acceptable Cronbach's alpha of 0.722. This study revealed varied severities of dysmenorrhea among the students as severe, moderate, and mild (45.6%, 39.8%, 14.6%, respectively). The data indicate that 30.8% of those with severe dysmenorrhea required medical attention and pain killer or herbs. The severity of dysmenorrhea differs in local and international studies [3, 9, 17]. The findings of severe dysmenorrhea among students in this study (30.8%) are similar to the reported findings in local studies among students in AlJouf University and King Abdulaziz University (34% and 29%, respectively) [3, 9]. It is also similar to the reported finding among adolescent students in Iraq and slightly higher than Jordan (32.9% and 19.85%, respectively) [17, 18].

In contrast, a study conducted in Greece showed a higher percentage of severe dysmenorrhea among nursing students (52.5%) than the present study [19]. Variations in dysmenorrhea severity may be connected to cultural differences in pain awareness and the subjects’ pain threshold, as described in a previous study linking ethnicity and pain perception [20]. In addition, the various scales used to evaluate the degree of dysmenorrhea and the different target populations may contribute to the different percentages.

Depressive symptoms affect menstrual cycle function and dysmenorrhea. In addition, menarche occurs during puberty, a developmental period with an increased risk of depressive symptoms and/or anxiety [12, 21]. This study found a significant association between dysmenorrhea and depression with dysmenorrhea being a predictor of depression and vice versa. Menstrual pain’s working effect and severity as well as the number of painful locations and days all correlated significantly and positively with depression. This may be because our target population was university students, who are more prone to mental disorders [22]. A recent study reported an association between gender and mental disorders. They found that the risk of acquiring major depressive symptoms, chronic pain conditions, and anxiety was higher in women than men [23]. Depressive symptoms may be related to females’ hormone fluctuations [24].

Furthermore, the misdiagnosis of mood disorders—such as PMDD (premenstrual dysphoric disorder) for depression—may underlie this correlation and increase the severity of dysmenorrhea [25]. Similarly, a study conducted in Georgia identified a significant relationship between primary dysmenorrhea and depression. It also found that women with mental disorders tend to have lower pain thresholds [26]. Moreover, another study reported high depression and anxiety scores due to the interference of dysmenorrhea with daily activities [27].

The mean age of the female students in this study was 20.64 ± 2.38 years with a mean age of menarche of 12.83 ± 1.64 years. This study found that older females experienced significantly less severe dysmenorrhea including lower pain intensity and fewer painful days. This is consistent with previous results, described in a comprehensive review that emphasizes that younger females have a higher risk of dysmenorrhea [28]. Furthermore, this study found that the age of menarche significantly and negatively correlated with severe dysmenorrhea and depression. This indicates that a later pubertal onset decreased the likelihood of having dysmenorrhea and depression. Similarly, older age upon menarche is protective against dysmenorrhea [29, 30]. Similar results were found among AlJouf University students and high school students in Kuwait [3, 31]. This might be because of the increasing contractility of uterine muscles due to the long and early introduction to uterine prostaglandins [32]. The release of prostaglandin leads to blood vessels contraction which cause abnormal uterine contractions, ischemia, and finally the increasing sensitivity of nerve endings. Moreover, it is thought that being exposed to this cycle from a young age contributes to the increasing severity of primary dysmenorrhea [33].

A noteworthy finding in our study were significant predictors of severe dysmenorrhea included pain killer and herb use for menstrual pain and the need for a doctor for menstrual pain. Although almost one-third of the students in this study had severe dysmenorrhea, reflected by painkillers and herbs being used by 47.8% and 48.7%, respectively, only 19.1% had visited a doctor for the pain. This finding is confirmed by previous studies conducted in Saudi Arabia, Iran, and Iraq [17, 34, 35]. Potential explanations include the tendency to normalize menstrual pain and being embarrassed to express menstrual experiences, which can lead to self-medicating and limit seeking professional help [36, 37].

Moreover, two-thirds of the females in this study had a regular menstrual cycle with a mean duration of 6.53 ± 1.60 days. Menstrual cycle regularity and duration were significant protective factors for depression. Severe depression and stress were linked to irregular and short-length menstrual cycles [38]. This could be due to the dysregulation of the hypothalamic–pituitary–adrenal (HPA) axis associated with depression and stress, as well as its influence on gonadotropin-releasing hormone (GnRH) [39, 40].

One of the limitations of the current study is that data were collected using a self-administrated online survey and a convenient nonprobability sampling technique due to COVID-19 pandemic restrictions and, therefore, is susceptible to recall bias. In addition, although PNU is the largest female university in the world, conducting the study at only one university might limit the generalizability of the results. In addition, students were assessed for depressive symptoms during the last 2 weeks by the PHQ-9, however, they were not asked whether they had menses during the last 2 weeks or not. This reflects the need to elaborate the temporal relationship between depression and the menstrual cycle among the students. Finally, the present study is cross-sectional and, as such, cannot demonstrate a cause–effect relationship between dysmenorrhea and its risk factors.

## Conclusions

Based on these findings, we conclude that severe dysmenorrhea is not an uncommon problem among Saudi university students. More attention should be paid to the proven predictors of severe dysmenorrhea including younger age, earlier menarche onset, medication usage, doctor visit for menstrual pain, and depression. The irregularity and short duration of the menstrual cycle also need attention due to its association with depression. Therefore, the findings of this study support the importance of increasing awareness of the management of painful menstruation, and the early detection to prevent future complications. There is, therefore, a definite need for public health campaigns delivering the correct information regarding these issues. Applying topical heat, exercise, and consuming diet rich in omega 3 and vitamin B are suggested for women with dysmenorrhea to improve the symptoms [41]. There is a need to consider the effect of painful menstruation on mental well-being and to adopt a multidisciplinary approach to women’s health.

## Data Availability

The data sets used and/or analysed during the current study are available from the corresponding author on reasonable request.

## Abbreviations

PNU: Princess Nourah bint Abdulrahman University
PHQ-9: Patient Health Questionnaire
WaLIDD: Working ability, location, intensity, days of pain, dysmenorrhea
DSM-5: The Diagnostic and Statistical Manual of Mental Disorders, fifth edition
